# Vinorelbine, cyclophosphamide and 5-FU effects on the circulating and intratumoural landscape of immune cells improve anti-PD-L1 efficacy in preclinical models of breast cancer and lymphoma

**DOI:** 10.1038/s41416-018-0076-z

**Published:** 2018-04-26

**Authors:** Stefania Orecchioni, Giovanna Talarico, Valentina Labanca, Angelica Calleri, Patrizia Mancuso, Francesco Bertolini

**Affiliations:** 1 Laboratory of Hematology-Oncology and Hemo-Lympho Pathology Unit, European Institute of Oncology, Milan, Italy; 2 Hemo-Lympho Pathology Unit, European Institute of Oncology, Milan, Italy

**Keywords:** Cancer microenvironment, Cancer models

## Abstract

**Background:**

Anti-PD-1 and anti-PD-L1 checkpoint inhibitors (CIs) are clinically active in many types of cancer. However, only a minority of patients achieve a complete and/or long-lasting clinical response. We studied the effects of different doses of three widely used, orally active chemotherapeutics (vinorelbine, cyclophosphamide and 5-FU) over local and metastatic tumour growth, and the landscape of circulating and tumour-infiltrating immune cells involved in CI activity.

**Methods:**

Immunocompetent Balb/c mice were used to generate models of breast cancer (BC) and B-cell lymphoma. Vinorelbine, cyclophosphamide and 5-FU (alone or in combination with CIs), were given at low-dose metronomic, medium, or maximum tolerable dosages.

**Results:**

Cyclophosphamide increased circulating myeloid derived suppressor cells (MDSC). Vinorelbine, cyclophosphamide and 5-FU reduced circulating APCs. Vinorelbine and cyclophosphamide (at medium/high doses) reduced circulating Tregs. Cyclophosphamide (at low doses) and 5-FU (at medium doses) slightly increased circulating Tregs. Cyclophosphamide was the most potent drug in reducing circulating CD3+CD8+ and CD3+CD4+ T cells. Vinorelbine, cyclophosphamide and 5-FU reduced the number of circulating B cells, with cyclophosphamide showing the most potent effect. Vinorelbine reduced circulating NKs, whereas cyclophosphamide and 5-FU, at low doses, increased circulating NKs. In spite of reduced circulating T, B and NK effector cells, preclinical synergy was observed between chemotherapeutics and anti-PD-L1. Most-effective combinatorial regimens where associated with neoplastic lesions enriched in B cells, and, in BC-bearing mice (but not in mice with lymphoma) also in NK cells.

**Conclusions:**

Vinorelbine, cyclophosphamide and 5-FU have significant preclinical effects on circulating and tumour-infiltrating immune cells and can be used to obtain synergy with anti-PD-L1.

## Introduction

Checkpoint inhibitors (CIs) have recently shown a remarkable clinical activity in a variety of types of cancer, but so far only a minority of patients treated with CIs alone has achieved a complete response and/or a long-lasting clinical benefit.^1–4^ As shown by some preclinical studies, the addition of clinically active targeted drugs to CIs might increase their in vivo activity, and some clinical studies are already investigating this hypothesis.^5–7^

Several preclinical studies (reviewed in refs.^8–10^) have suggested that some chemotherapy drugs can (re)activate tumour targeting immune responses. The present preclinical study had three aims: a) to compare systematically by multiparametric flow cytometry the dosage-dependent and time-dependent effects of three different chemotherapeutic drugs over a wide panel of circulating immune cells including effectors, suppressors, regulatory and antigen-presenting cells; b) to investigate a possible synergy between these drugs and CIs anti-PD-1 and anti-PD-L1; c) to compare systematically the effects of these chemotherapeutics—alone or in combination with CIs—over the landscape of infiltrating, intratumoural immune cells.

Considering a possible long-term combinatorial therapeutic use of chemotherapy drugs along with CIs, we selected three drugs which can be administered orally (either in a continuous, low-dose metronomic fashion, see ref.^11^, or at higher doses) and have a favourable toxicity profile, namely vinorelbine (V), cyclophosphamide (C) and 5-FU, used in this study to mimic the orally active analogue capecitabine.

To possibly avoid model-related biases, we studied two different preclinical models of cancer, namely triple negative breast cancer (BC, by means of a validated orthotopic model based upon the injection of murine 4T1 cells in the mammary fat pad followed by mastectomy and the study of subsequent lung metastases, see refs.^12–14^), and B cell lymphoma (by means of sc injection of murine A20 cells, see ref.^5^).

## Materials and methods

### Cell cultures

The 4T1 BC cell line and the A20 B cell lymphoma cell line were purchased from ATCC, (Manassas, VA, USA), expanded and stored according to the producer’s instructions. Cells were tested and authenticated by the StemElite ID System (Promega, Fitchburg, WI, USA). Cells were tested every six months for Mycoplasma by means of the ATCC Universal Mycoplasma Detection Kit 30–1012, cultured for no more than two weeks and used for no longer than 15 passages.

### Xenografts

Experiments involving animals were approved by the Italian Ministry of Health and have been done in accordance with the applicable Italian laws (D.L.vo 26/14 and following amendments), the Institutional Animal Care and Use Committee and the institutional guidelines at the European Institute of Oncology. In vivo studies were carried out in immune-competent BALB/cOlaHsd female mice (Envigo, UK) and in immunodeficient NSG mice (Charles River, Italy), 6–9-weeks old. Mice were bred and housed under pathogen-free conditions in the animal facilities at the European Institute of Oncology–Italian Foundation for Cancer Research (FIRC) Institute of Molecular Oncology (IEO–IFOM, Milan, Italy) campus.

To generate syngeneic models of BC^11–13^ and of non-Hodgkin’s lymphoma^5^ in BALB/c and NSG mice, 0.1 × 10^6^ 4T1 triple negative BC cells or 5 × 10^6^ A20 B cell lymphoma cells were injected in the mammary fat pad (4T1, ref.^11–13^) and subcutaneously into the right flank (A20, ref.^5^), respectively. Tumour growth was monitored weekly using digital callipers, and tumour volume was calculated according to the formula: *L* × *W*^2^/2 = mm^3^, where *W* represents the width and *L* the length of the tumour mass.

### BC metastasis model

In separate studies, BC resection was done 25 days after tumour implant, as previously described.^11–13^ Fifteen days after mastectomy, mice were sacrificed by carbon dioxide inhalation and lung tissues were removed. To confirm the presence of metastases, sections were cut and stained with haematoxylin and eosin (H&E), as previously described.^11–13^ In brief, lungs were fixed in 4% phosphate-buffered formalin and embedded in paraffin. Five micrometres thick sections of lungs were made, and slides were counterstained with H&E for the detection of metastases. Images were acquired with a ScanScope XT scanner (Leica, Germany) and analysed with Aperio Digital Pathology software.

### In vivo therapy

Tumour-bearing mice and tumour-free mice (*n* = 5 per study arm) were treated with either vehicle or with different drugs used as single agents or in combination. Drug dosages were based on literature data^14, 15^ as representative of a spectrum encompassing oral low-dose metronomic, medium, or maximum tolerable dosages, and associated with no or acceptable toxicity, as well as no significant changes in mouse weight. Blood was collected weekly from the tail vein, and circulating immune cells were determined by multiparametric, 10-colour flow cytometry. C was given ad libitum through the drinking water to administer an approximate dose of 5 (low dose),10, 20 (medium doses) and 40 (high dose) mg/kg/day, based on the estimated daily consumption of 2 ml for a 20 g mouse, as previously described.^13^ V (3 mg/kg for low dose, 6 and 9 mg/kg for medium doses, 12 mg/kg for high dose) and 5-FU (5 mg/Kg for low dose,10–25 mg/Kg for medium doses, 50 mg/kg for high dose) were dissolved in saline and administrated 3 times a week for 3 weeks by oral gavage (V) or by intraperitoneal injection (5-FU). Anti-PD-L1 (10 F.9G2, Bioxcell, West Lebanon, NH, USA) and anti-PD-1 (J43, Bioxcell) or rat IgG2b isotype control (LTF-2, Bioxcell) (0.2 mg/mouse) were dissolved in PBS and given intraperitoneally every 2 days for a total of 5 doses.

### Tumour dissociation for the profiling of cell surface markers

Mice were either sacrificed after 35 days from tumour injection (A20 model) or the tumour was removed after 25 days (4T1 model) to generate a single cell suspension. Briefly, after mechanical dissociation with gentle MACS Dissociator (Miltenyi Biotech, Germany), tumours were placed in culture medium (1:1 of Dulbecco’s Modified Eagle’s Medium with high glucose and Ham’s F-12 Nutrient Mixture, EuroClone, UK) supplemented by 2 mg/mL collagenase (Sigma) and 0.1 mg/mL DNase I (Qiagen, the Netherlands), and digested for 1–2 h at 37 °C. A single cell suspension was obtained by sequential dissociation of the fragments by gentle pipetting, to further disintegrate cell clumps, followed by filtration through a 100-μm nylon mesh.

### Flow cytometry

At least 500,000 cells per sample were acquired using a 3-laser, 10-colour flow cytometer (Navios, Beckman Coulter, Brea, CA, USA). As reported in supplementary Tab. 1, viable cells (negative for 7-aminoactinomycin, 7AAD) were labelled with a panel of antibodies (Beckman Coulter or BD Biosciences, San Diego, CA, USA) to analyse immune cell populations. Lymphocytes and myeloid cells were characterised using state-of-the-art markers.^16–19^ MDSCs were identified as recently decribed.^18^ Specifically, cells were gated for size, singlets and then by positive and negative markers: CD3+CD4+ and CD3+CD8+ T cells, CD335+ NKs, CD19+ B cells, CD3+CD4+CD25++CD127^low/neg^ Tregs, Gr1-CD11b+CD11c+ monocytes, SSC^low^CD11b+Gr1+ MDSC (monocytic and granulocytic), SSC^high^CD11b+Gr1+ granulocytes and CD11c+CD11b-Gr1- antigen-presenting cells (Suppl. Table 1).

### Immunohistochemistry

Tumour mass were removed, fixed in 4% phosphate-buffered formalin and embedded in paraffin. For immunohistochemical analysis, 3–4 μm-thick tissue sections were stained with primary antibody CD31 (clone JC70A, DAKO). Antigen retrieval was performed with PTlink (Dako Cytomation, Glostrup, Denmark; code PT100/PT101) and the EnVision Flex Target Retrieval Solution High pH (DakoCytomation; code K8004). The immunohistochemical staining procedure was performed using a Dako Autostainer (Dako, Glostrup, Denmark). Images were acquired with a ScanScope XT scanner (Leica) and analysed with Aperio Digital Pathology software.

### Statistical analysis

The Shapiro-Wilk test was used to assess normality. Most data were not normally distributed so all statistical comparisons used the nonparametric Mann–Whitney *U*-test of. All *p* values are two sided. Differences were considered significant for *p* < 0.05 after Bonferroni correction. The statistical analyses were performed with GraphPad Prism software.

## Results

### V, C and 5-FU effects on subsets of circulating immune cells

**Fig. 1:**
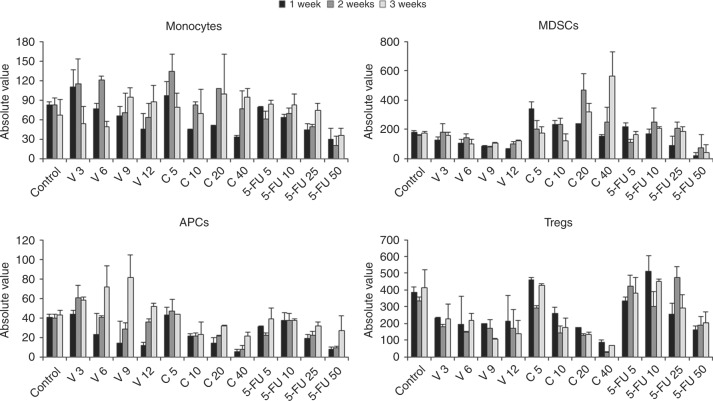
Total numbers of monocytes, MDSCs, APCs and Tregs in mice treated with different types and dosages of chemotherapy drugs. BALB/c mice (*n* = 5 per study arm) were treated with different dosages of V, C and 5-FU and bleed weekly for 3 weeks to enumerate total numbers of circulating monocytes (top panel on the left), MDSCs (top panel on the right), APCs (bottom panel on the left) and Tregs (bottom panel on the right). MDSCs are shown as the total of monocytic-MDSCs (about 10% of all MDSCs) and granulocytic-MDSCs (about 90% of the total population)

**Fig. 2:**
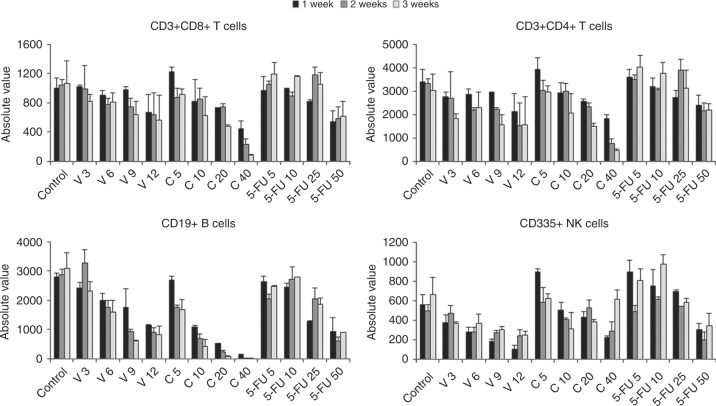
Total numbers of T, B and NK cells in mice treated with different types and dosages of chemotherapy drugs. BALB/c mice (*n* = 5 per study arm) were treated with different dosages of V, C and 5-FU and bleed weekly for 3 weeks to enumerate total numbers of circulating CD3+CD8+ T cells (top panel on the left), CD3+CD4+ T cells (top panel on the right), B cells (bottom panel on the left) and NK cells (bottom panel on the right)

As shown in Figs. 1–2, Suppl. Figure 1 and Suppl. Tab. 2, the administration of V, C and 5-FU at different dosages was associated with profound effects on the landscape of circulating immune cells in tumour-free recipient BALB/c mice, evaluated as in Suppl. Figure 2. Suppl. Figure 1 shows the effect of four different dosages of these drugs on the total number of CD45+ murine white blood cells (WBCs) and on granulocytes during 3 weeks. When considering all WBCs, V and C at medium-high dosages were associated with a significant reduction in the number of circulating cells. 5-FU reduced the total number of CD45+ white cells only at higher dosages. When considering granulocytes, V at all dosages significantly reduced these cells, C reduced them only at medium and high doses and 5-FU only at the highest investigated dosage.

Figure 1 shows the effect of V, C and 5-FU on circulating levels of immune cells associated with suppressors, regulatory and APC functions. V and C, at low and medium dosages, induced an increase in the amount of circulating monocytes, in particular during the second week of administration. 5-FU, at high doses, reduced the number of circulating monocytes.

Values of circulating MDSCs are shown as the total of monocytic-MDSCs (about 10% of all MDSCs) and granulocytic-MDSCs (about 90% of the total population). C induced an increase in MDSCs count, notably in the first week of administration when given at low doses and at the second or third week of administration when given at higher doses. V, C and 5-FU, when given at high doses, reduced the number of circulating APCs in the first week of administration, whereas a trend toward higher values of circulating APCs was observed after two weeks of administration of V at medium doses. V at medium doses, and C at medium and high doses, reduced the number of circulating Tregs. At variance, C at low doses and 5-FU at medium doses slightly increased the number of circulating Tregs.

Figure 2 shows the effect of V, C and 5-FU on circulating levels of immune cells associated with effector activity. C, at high dosage, significantly reduced circulating CD3+CD8+ effector T cells. V slightly reduced CD3+CD4+ T cells after two weeks of administration. C, at high doses, more significantly decreased CD3+CD4+ T cells. V, C and 5-FU reduced circulating CD19+ B cells, with C showing the most significant effect at medium and high doses. Regarding CD335+ NK cells, V at medium and high doses reduced their number, whereas the administration of C and 5-FU at low doses was associated with an increase in the number of circulating NKs.

We also investigated the effect of V, C and 5-FU on the circulating immune cell atlas in BC 4T1-bearing mice (Suppl. Figure 3-8). At variance with tumour-free mice, tumour-bearing mice had an inflammatory reaction that paralleled tumour growth, so that in the second and third week after tumour injection all myeloid and lymphoid cells were significantly increased in both untreated and treated mice. In spite of this inflammatory reaction, in tumour-bearing mice we observed almost all the same trends observed in tumour-free mice, with few exceptions. In tumour-bearing mice, C did not increase circulating monocytes, MDSCs and Tregs. Circulating B cells were reduced by C at higher doses, but not by V and by 5-FU at low and medium doses. V and C slightly reduced NK cells. These data in tumour-bearing mice should be interpreted with caution, because of the extent of the inflammatory reaction which is not always present in human patients with limited-stage neoplastic diseases.

### Preclinical efficacy and intratumoural immune cell landscape in a BC model treated with V, C and/or 5-FU with or without CIs

**Fig. 3:**
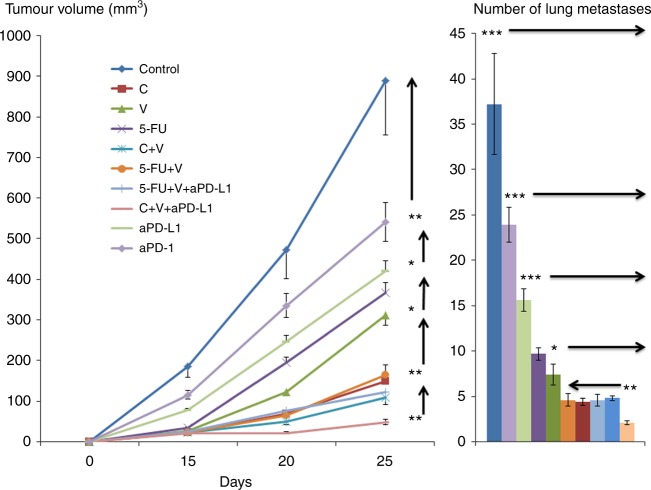
Local and metastatic BC tumour growth in mice treated with checkpoint inhibitors and/or different types of chemotherapy drugs. To generate syngeneic models of BC in BALB/c mice, 0.1 × 10^6^ 4T1 triple negative BC cells were injected in the mammary fat pad. Tumour growth was monitored weekly (left panel). In separate studies, BC resection was done 25 days after tumour implant, 15 days after mastectomy mice were sacrificed and lung tissues were removed. To confirm the presence of metastases, sections were cut, stained and investigated for the detection of metastases (right panel). Mice were treated with checkpoint inhibitors anti-PD-1 or anti-PD-L1 and/or different dosages of V, C and 5-FU. (*n* = 5 per study arm; *p < 0.05, **p < 0.01, ***p < 0.001)

Immune-competent BALB/c mice were orthotopically injected with murine triple negative 4T1 BC cells. Figure 3 shows local (left panel) and post-mastectomy, metastatic tumour growth (right panel) in mice treated with V, C and/or 5-FU with or without CIs. Anti-PD-L1 was significantly more effective than anti-PD-1 in this model, and for this reason anti-PD-L1 was the CI used in combinatory studies. V, C and 5-FU were used at the dosages of 9, 20, and 50 mg/Kg, respectively. These dosages were selected as higher dosages were found to be too much toxic when used in combination (Orecchioni et al., unpublished observations;^14, 20^).

When compared to untreated controls, V, C and 5-FU were effective in reducing local and metastatic tumour growth. C was slightly more effective than the other two drugs. Combinatorial regimens associating C, 5-FU and anti-PD-L1 did not add to the preclinical activity of C alone. The association of V, C and anti-PD-L1 was the most effective combinatorial regimen in terms of local and metastatic BC control.

**Fig. 4:**
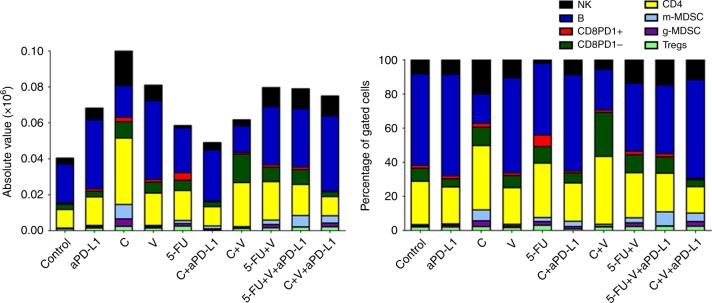
Atlases of intratumoural infiltrates in mice injected with BC cells and treated with checkpoint inhibitors and/or different types of chemotherapy drugs. To generate syngeneic models of BC in BALB/c mice, 0.1 × 10^6^ 4T1 triple negative BC cells were injected in the mammary fat pad, and BC resection was done 25 days after tumour implant. Mice were treated with checkpoint inhibitors anti-PD-1 or anti-PD-L1 and/or different dosages of V, C and 5-FU. The panel on the left shows the total numbers of different immune cells infiltrating the tumours as enumerated by flow cytometry. The panel on the right shows the percentages of different immune cell populations. (*n* = 5 per study arm)

Figure 4 shows the total number and the percentage of intratumoural immune cells in local 4T1 BC neoplastic lesions at mastectomy on day 25. When compared to untreated controls, anti-PD-L1 treatment alone was associated with an increase in infiltrating immune cells, and in particular of B cells. In spite of a reduction in circulating WBCs, treatment with C alone was associated with the largest tumour infiltration by NK, CD3+CD4+ T cells and m-MDSCs, and with the lowest B cell infiltration. The combinatorial treatment of V+C was associated with the largest CD8+PD-1^negative^ count, abundant CD3+CD4+ T cells and low Treg counts. C, but not V, 5-FU and anti-PD-L1, significantly increased intratumoural activated CD8+CD25+CD69+ T cells (Suppl. Fig. 9). The combinatorial treatment with C+V+anti-PD-L1 was associated with the largest intratumoural B cell count and the lowest number of CD3+CD8+PD-1+ T cells. As shown in Suppl. Fig. 10, an investigation of the clonality of B cells infiltrating 4T1 BC tumours indicated that these cells are likely to be polyclonal. As reported in Suppl. Fig. 11, immunohistochemistry studies shown that C, but not V and 5-FU, significantly reduced microvessel density in 4T1 tumours.

To determine if the antitumour effect of chemotherapy was dependent—at least in part—on the immune system, we also investigated 4T1-bearing immunodeficient NSG mice (Suppl. Fig. 12). In this setting, C was the most active chemotherapeutic. The synergistic activity of the addition of V to C was not observed. This suggest that, in immunocompetent mice, this synergy might be due to some of the V effects on immune cells.

### Preclinical efficacy and intratumoural immune cell landscape in a B cell lymphoma model treated with V and/or C, with or without CIs

**Fig. 5:**
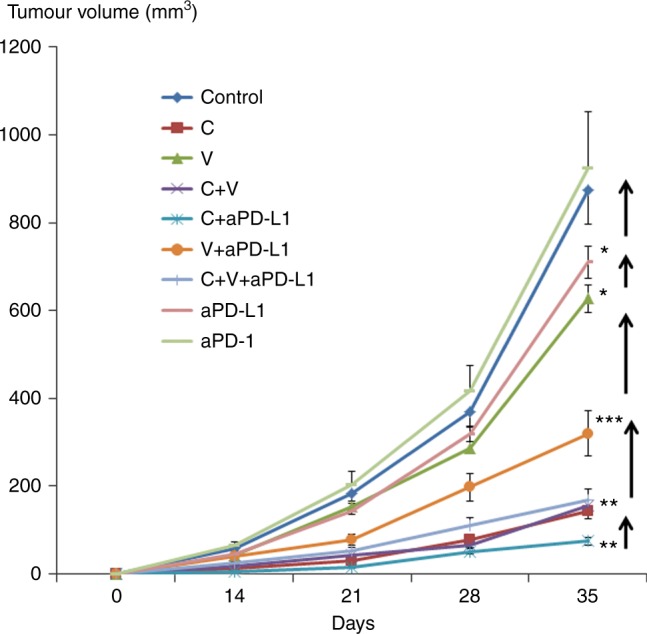
B cell lymphoma growth in mice treated with checkpoint inhibitors and/or different types of chemotherapy drugs. To generate syngeneic models of B-cell lymphoma in BALB/c mice, 5 × 10^6^ A20 B cell lymphoma cells were injected subcutaneously into the right flank. Tumour growth was monitored weekly. Mice were treated with checkpoint inhibitors anti-PD-1 or anti-PD-L1 and/or different dosages of V or C. (*n* = 5 per study arm; *p < 0.05, **p < 0.01, ***p < 0.001)

Immune-competent BALB/c mice were injected sc with murine A20 B cell lymphoma cells. Figure 5 shows tumour growth in mice treated with V and/or C with or without CIs. As in the BC model, anti-PD-L1 was significantly more effective than anti-PD-1 in this lymphoma model, and for this reason anti-PD-L1 was the CI used in combinatory studies. 5-FU was not used in this model because it is not clinically active in human B cell lymphoma. V and C were used at the dosages of 9 and 20 mg/Kg, respectively. Again, these dosages were selected as higher dosages were found to be too much toxic when used in combination.

When compared to untreated controls, V was only marginally effective in reducing lymphoma growth, and C was significantly more effective. The association of C and anti-PD-L1 was the most effective combinatorial regimen in terms of disease control.

**Fig. 6:**
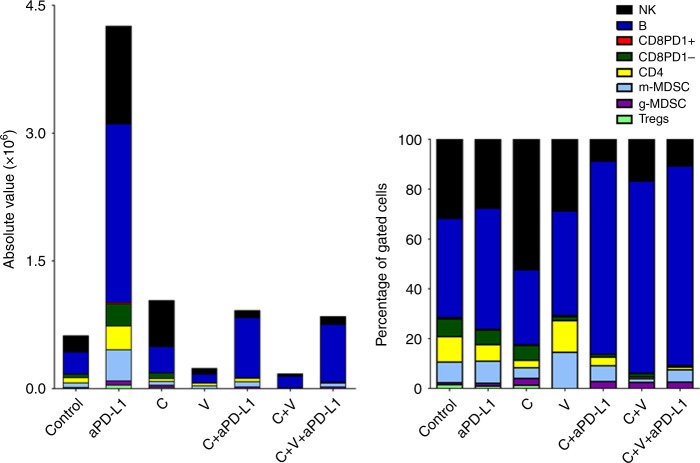
Atlases of intratumoural infiltrates in mice injected with B cell lymphoma and treated with checkpoint inhibitors and/or different types of chemotherapy drugs. Mice were treated with checkpoint inhibitors anti-PD-1 or anti-PD-L1 and/or different dosages of V or C. To generate syngeneic models of B-cell lymphoma in BALB/c mice, 5 × 10^6^ A20 B cell lymphoma cells were injected subcutaneously into the right flank. The panel on the left shows the total numbers of different immune cells infiltrating the tumours as enumerated by flow cytometry. The panel on the right shows the percentages of different immune cell populations. (*n* = 5 per study arm)

Figure 6 shows the total number and the percentage of intratumoural immune cells in neoplastic lesions at sacrifice on day 35. When compared to untreated controls, anti-PD-L1 treatment alone was associated with the largest increase in the total number of infiltrating immune cells, and in particular in B cells (different in size and forward scatter from A20 lymphoma cells). Treatment with C alone—when compared to controls—was associated with an increase in NK tumour infiltration. Treatment with V decreased the immune cell infiltrate. The addition of anti-PD-L1 to V and C were associated with an increase in B cell count.

## Discussion

The first aim of the present study was to compare systematically in immune-competent mice the dosage and time-dependent effects of different chemotherapeutic drugs over a wide panel of circulating immune cells. We selected a panel of drugs as representative of three different classes of oral chemotherapeutics, all of which known to have a convenient toxicity profile, direct effects on cancer cells and parallel effects on several immune cell populations.^9, 10, 14,]^ We were particularly interested in investigating the lower/metronomic dosages, associated with a specific impact on immune cells and a lower toxicity that renders combinatorial regimens more possible in clinical trials.^21^

Vinorelbine, a vinca alkaloid, was known to promote polyploidisation and to interfere with mitosis. These two effects were reported in the past to enhance the immune recognition of cancer cells by the immune system when V was administered with cisplatin, possibly by reducing Treg activity. Notably, not all vinca alkaloids share these effects on immune cells. In fact, V and vinblastine targets immune cells more effectively than vincristine.^9, 21, 22^ C, an alkylating agent, was known to induce inter-strand or intra-strand DNA crosslinks able to destabilise DNA during replication, to deplete Tregs, to expand NK cells and MDSCs, and to promote several T cell-dependent immune responses against cancer.^23–26^ The antimetabolite 5-FU was known to inhibit the synthesis of nucleic acids, to increase the frequency of cancer-infiltrating T cells and to deplete MDSCs.^27^

Our present data expand the knowledge on the effects of these three chemotherapeutics over the circulating immune cell landscape in mice. After 2 weeks of administration, V and C at low and medium dosages increased the count of circulating monocytes. At variance, higher doses of 5-FU reduced the number of circulating monocytes. Monocytes seems to be important in the context of checkpoint inhibition because recent data indicate that anti-PD-1 monoclonal antibodies can be captured within minutes from the T cell surface by PD-1^−^ tumour-associated macrophages.^28^ We are investigating whether chemotherapeutics might have an effect over this detrimental antibody capture.

V, C and 5-FU reduced the number of circulating APCs in the first week of administration, whereas medium doses of V increased the number of circulating APCs after two weeks of administration. These data are relevant as APC crosstalk with T and NK effectors play a crucial role in the modulation of anti-tumour immunity.^29^ Tregs were reduced by higher dosages of V, C and 5-FU, whereas, unexpectedly, low and medium dosages of C and 5-FU slightly increased the number of circulating Tregs. A recent paper has suggested that Treg depletion may potentiate checkpoint inhibition in the claudin-low BC subtype.^30^ Therefore, in combinatorial regimens with CIs, the dosages of V, C and 5-FU should be careful evaluated. C was the most potent drug in reducing the number of circulating CD3+CD8+ and CD3+CD4+ T cells. All investigated chemotherapeutics reduced the number of circulating B cells, with C showing the most profound effect. As for NK cells, we observed that V reduced their number, at variance with C and 5-FU that, at low doses, increased NK number in the peripheral blood.

The second aim of the study was to investigate a possible synergy between the three chemotherapy drugs and the CIs anti-PD-1 and anti-PD-L1. In spite of the significant reduction of circulating effectors such as CD3+CD8+ and CD3+CD4+ T cells, B and NK cells, in the present preclinical study we observed a synergy between chemotherapeutics and the CI anti-PD-L1 in two immune-competent models of cancer, namely local and metastatic triple negative BC and B cell lymphoma. As the clinical experience indicates that in some patients resistance can occur after therapy with CIs, we are generating the appropriate models and planning studies to understand what therapy/sequence with chemotherapeutics can overcome drug resistance to CIs.

The third aim of the study was to compare systematically by multiparametric flow cytometry the effects of these chemotherapeutics, alone or in combination with CIs, over the landscape of infiltrating, intratumoural immune cells. Unexpectedly, we observed that the differences induced in the peripheral blood by chemotherapeutics were not paralleled intratumourally. When compared to untreated controls, the most effective combinatorial regimens (V+C+anti-PD-L1 in BC and C+anti-PD-L1 in lymphoma) where associated with neoplastic lesions enriched in B cells. In BC-bearing mice (but not in mice with B cell lymphoma), the most effective combinatorial regimen was also significantly enriched in NK cells.

Differences in the total amount of infiltrating, intratumoural immune cells between the two models might be due to the different immunogenicity of chemotherapy-treated BC cells vs. B cell lymphoma, and/or to the different site of tumour growth, i.e., the mammary fat pad and the lung for BC and the sc tissues for the B cell lymphoma.^5, 11–13, 15, 19^

It has been reported in the past that in preclinical lung adenocarcinoma models immunogenic chemotherapies based upon C and oxaliplatin can sensitise lung adenocarcinomas to CIs that were otherwise not effective.^29^ Similarly, in a preclinical model of *BRCA1*-mutated triple-negative BC (a disease frequently associated with increased somatic mutational load and large numbers of tumour-infiltrating lymphocytes), cisplatin treatment combined with two CIs substantially augmented antitumour immunity.^31^ In another study in a glioblastoma model, the preclinical efficacy of the CI anti-PD-1 was enhanced by local and abrogated by systemic chemotherapy based upon carmustine.^32^

Taken together, our data indicate that chemotherapeutics have very complex effects on the circulating landscape of immune cells, with subtle but significant differences related to the dosage and duration of the administration of the drugs. Notably, these effects on circulating immune cells differ qualitatively and quantitatively from those observed in the intratumoural immune cell infiltrate. The present data also suggest that oral chemotherapy might add to the effect of CIs, even though different types and sites of cancer generate significantly different atlases of intratumoural infiltrates. Several clinical trials are already investigating these combinatorial therapies.^33^ As the strength and timing of the anticancer response is influenced by a complex set of tumour, host and environmental factors including drug dosage and effects on cancer, immune, vascular and stromal cells,^8, 27, 34, 35^ a biology-centric^36^ design of future clinical trials combining chemotherapy and CIs should include the investigation of all these facets.

## Electronic supplementary material

Legends of supplementary figures

Suppl. Fig. 1

Suppl. Fig. 2

Suppl. Fig. 3

Suppl. Fig. 4

Suppl. Fig. 5

Suppl. Fig. 6

Suppl. Fig. 7

Suppl. Fig. 8

Suppl. Fig. 9

Suppl. Fig. 10

Suppl. Fig. 11

Suppl. Fig. 12

Suppl. Table 1

Suppl. Table 2

## Availability of data and material

Data and material are available upon request.
