# Supplementary material for: Vinorelbine, cyclophosphamide and 5-FU effects on the circulating and intratumoural landscape of immune cells improve anti-PD-L1 efficacy in preclinical models of breast cancer and lymphoma
Source: Br J Cancer. 2018 Apr 26;118(10):1329–36. doi: 10.1038/s41416-018-0076-z (PMC5959935; doi:10.1038/s41416-018-0076-z)
Supplement: Supplementary file 1 — Legends of supplementary figures [file 41416_2018_76_MOESM1_ESM.docx]

**Supplementary figure 1**

**Total numbers of total white blood cells and granulocytes in mice treated with different types and dosages of chemotherapy drugs.**

BALB/c mice (n=5 per study arm) were treated with different dosages of V, C and 5-FU as indicated in the bottom of the graphs and bleed weekly for three weeks to enumerate total numbers of circulating CD45+ white blood cells (top panel) and granulocytes (bottom panel).

**Supplementary figure 2**

***Representative gating strategy for the identification of mouse immune cells subsets***

**(A)** Morphological gates showing the exclusion of dead cells (cells positive for 7-AAD) and doublets (based on SSC-A vs. SSC-H parameters) applied before gating for CD45^+^ cells (based on SSC-H vs. CD45 parameters), both for tumour tissue and peripheral blood analysis. **(B)** Gating strategy used to define immune cell subpopulations in tumour mass of BALB/c mice: 1) CD3^-^CD19^+^ B cells and CD3^-^CD335^+^ NK cells, 2) distinct T cells subsets including cytotoxic T cells (CD3^+^CD8^+^) and T helper cells (CD3^+^CD4^+^), exhausted CD8^+^PD-1^+^ cells and regulatory T cells (CD4^+^CD25^++^CD127^low/neg^), and 3) dot plots showing distribution of m-MDSCs (CD11b^+^Ly6C^high^Ly6G^-^) and g-MDSCs (CD11b^+^Ly6C^low^Ly6G^+^). **(C)** Representative FACS profiles showing myeloid cells in peripheral blood of BALB/c tumour-free mice: granulocytes (SSC^high^CD11b^+^Gr-1^+^), antigen-presenting cells (CD11b^-^Gr-1^-^CD11c^+^) , monocytes (CD11b^+^ Gr-1^-^CD11c^+^) and total-MDSCs (SSC^low^CD11b^+^Gr-1^+^).

**Supplementary figure 3**

**Total numbers of total white blood cells and granulocytes in 4-T1 BC bearing mice treated with different types and dosages of chemotherapy drugs.**

4-T1-bearing BALB/c mice (n=5 per study arm) were treated with different dosages of V, C and 5-FU and bleed weekly for three weeks to enumerate total numbers of circulating CD45+ white blood cells (top panel) and granulocytes (bottom panel).

**Supplementary figure 4**

**Total numbers of monocytes, MDSC, APCs and Tregs in 4-T1 BC bearing mice treated with different types and dosages of chemotherapy drugs.**

4-T1-bearing BALB/c mice (n=5 per study arm) were treated with different dosages of V, C and 5-FU and bleed weekly for three weeks to enumerate total numbers of circulating monocytes, MDSC, APCs and Tregs.

**Supplementary figure 5**

**Total numbers of CD3+CD8+ T cells, CD3+CD4+ T cells, B cells and NK cells in 4-T1 BC bearing mice treated with different types and dosages of chemotherapy drugs.**

4-T1-bearing BALB/c mice (n=5 per study arm) were treated with different dosages of V, C and 5-FU and bleed weekly for three weeks to enumerate total numbers of circulating CD3+CD8+ T cells, CD3+CD4+ T cells, B cells and NK cells.

**Supplementary figure 6**

**4-T1 BC tumour growth in mice treated with different V dosages**

4-T1-bearing BALB/c mice (n=5 per study arm) were treated with different dosages of V. (n=5 per study arm; * p<0.05, ** p<0.01, *** p<0.001).

**Supplementary figure 7**

**4-T1 BC tumour growth in mice treated with different C dosages**

4-T1-bearing BALB/c mice (n=5 per study arm) were treated with different dosages of C. (n=5 per study arm; * p<0.05, ** p<0.01, *** p<0.001).

**Supplementary figure 8**

**4-T1 BC tumour growth in mice treated with different 5-FU dosages**

4-T1-bearing BALB/c mice (n=5 per study arm) were treated with different dosages of 5-FU. (n=5 per study arm; * p<0.05, ** p<0.01, *** p<0.001).

**Supplementary figure 9**

**Intratumoural CD8+C25+CD69+ activated T cells**

4-T1-bearing BALB/c mice (n=5 per study arm) were treated with anti-PD-L1, V, C, or 5-FU. At sacrifice, intratumoural CD8+C25+CD69+ activated T cells were enumerated by flow cytometry. (n=5 per study arm; * p<0.05, ** p<0.01, *** p<0.001).

**Supplementary figure 10**

**Representative investigation of intratumoural CD19+ B cells**

Flow cytometric analysis of kappa and lambda light chain expression in polyclonal B cells infiltrating 4T1 BC tumours.

**Supplementary figure 11**

**Representative investigation of microvessel density**

4-T1-bearing BALB/c mice (n=5 per study arm) were treated with V, C, or 5-FU. At sacrifice, microvessel density was investigated by immunohistochemistry. Only C (but not V and 5-FU) significantly decreased the frequency of CD31+ intratumoural vessels.

**Supplementary figure 12**

**Local and metastatic BC tumour growth in immunodeficient NSG mice treated with different types of chemotherapy drugs.**

To generate syngeneic models of BC in NSG mice, 0.1 × 10^6^ 4T1 triple negative BC cells were injected in the mammary fat pad. Tumour growth was monitored weekly (left panel). In separate studies, BC resection was done 25 days after tumour implant, 15 days after mastectomy mice were sacrificed and lung tissues were removed. To confirm the presence of metastases, sections were cut, stained and investigated for the detection of metastases (right panel). Mice were treated with different dosages of V, C and 5-FU. (n=5 per study arm; * p<0.05, ** p<0.01, *** p<0.001).
