# Supplementary figures and images for: Vinorelbine, cyclophosphamide and 5-FU effects on the circulating and intratumoural landscape of immune cells improve anti-PD-L1 efficacy in preclinical models of breast cancer and lymphoma
Source: Br J Cancer. 2018 Apr 26;118(10):1329–36. doi: 10.1038/s41416-018-0076-z (PMC5959935; doi:10.1038/s41416-018-0076-z)

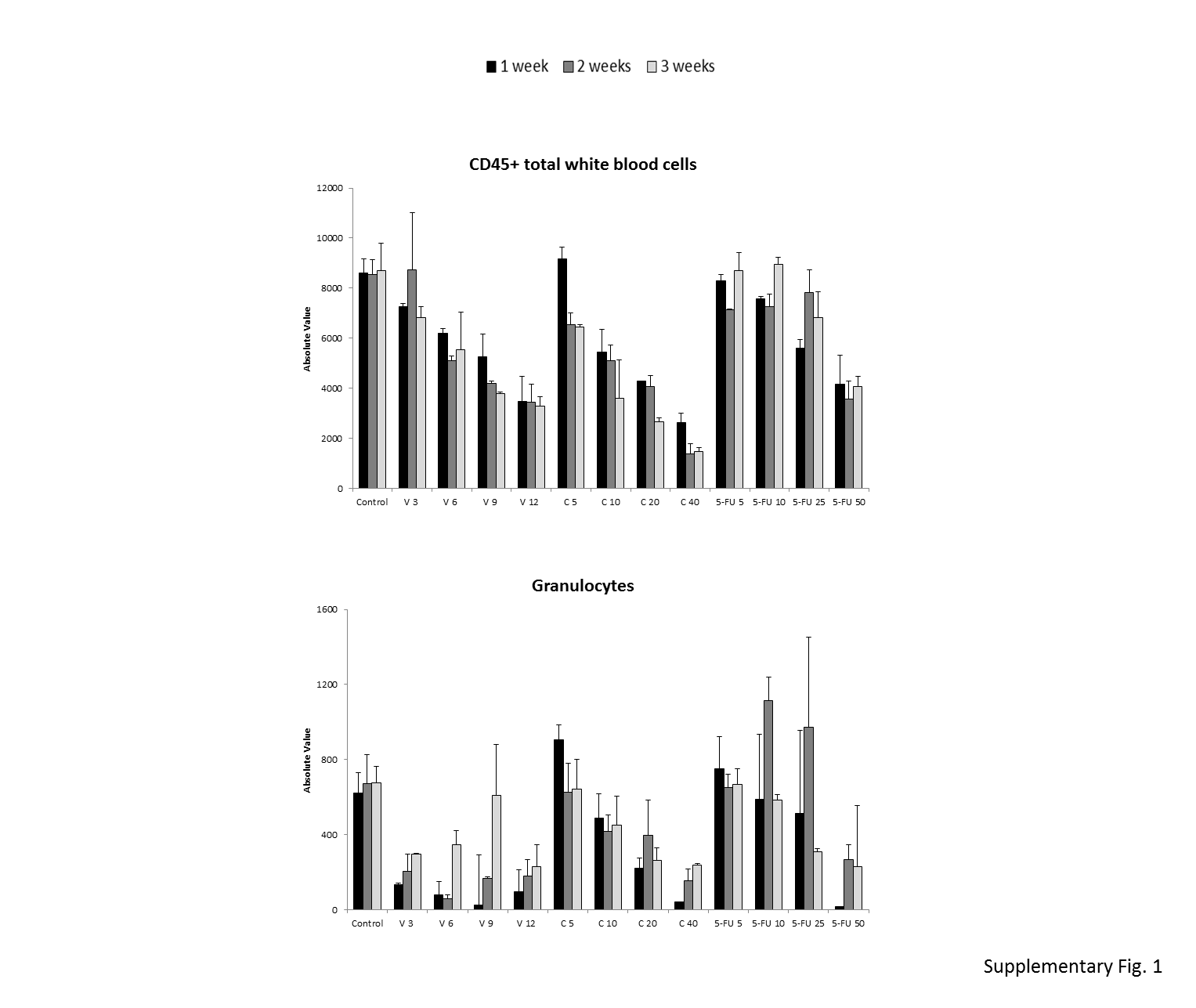

Supplement: Supplementary file 2 — Suppl. Fig. 1 [file 41416_2018_76_MOESM2_ESM.tif]

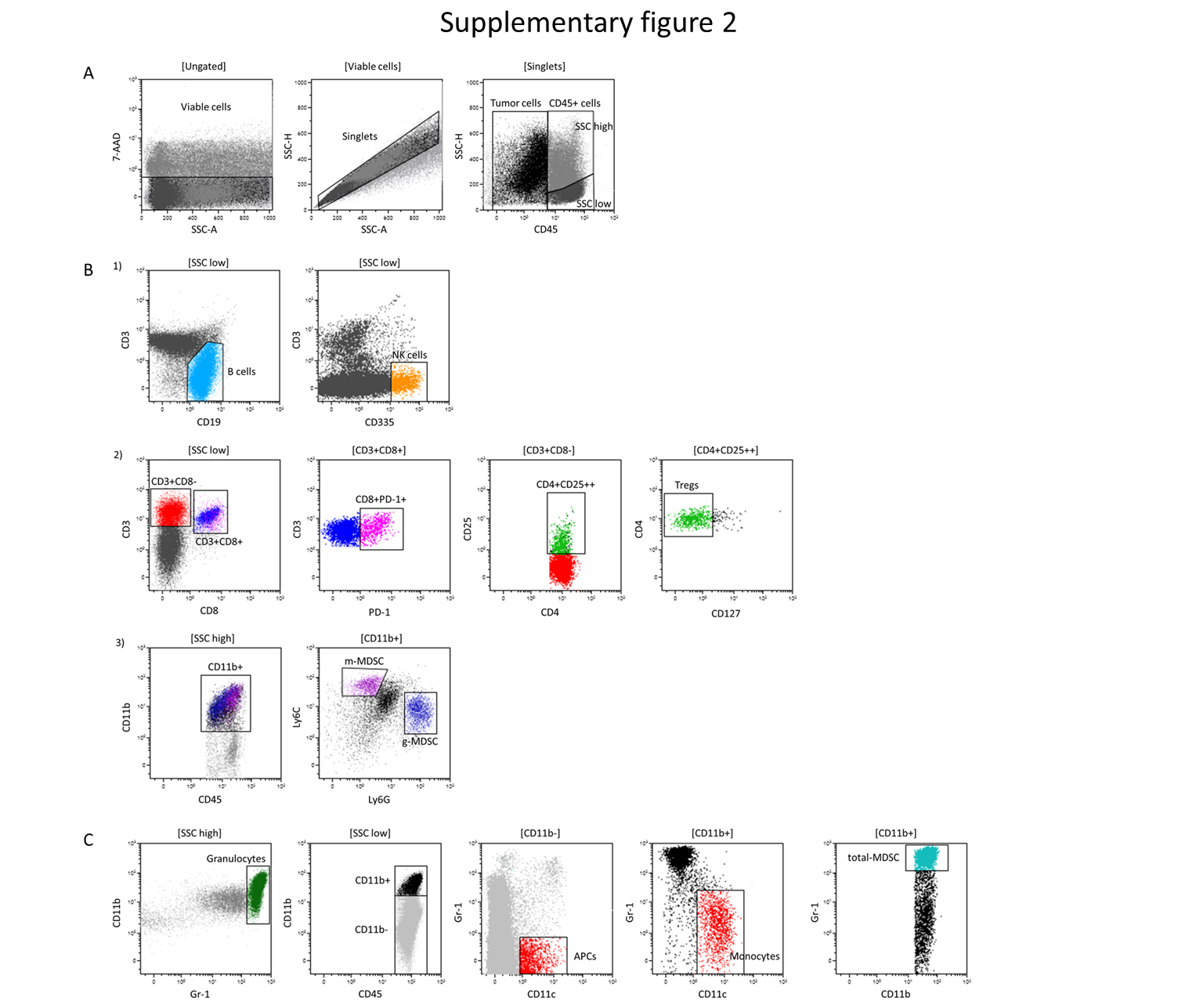

Supplement: Supplementary file 3 — Suppl. Fig. 2 [file 41416_2018_76_MOESM3_ESM.tif]

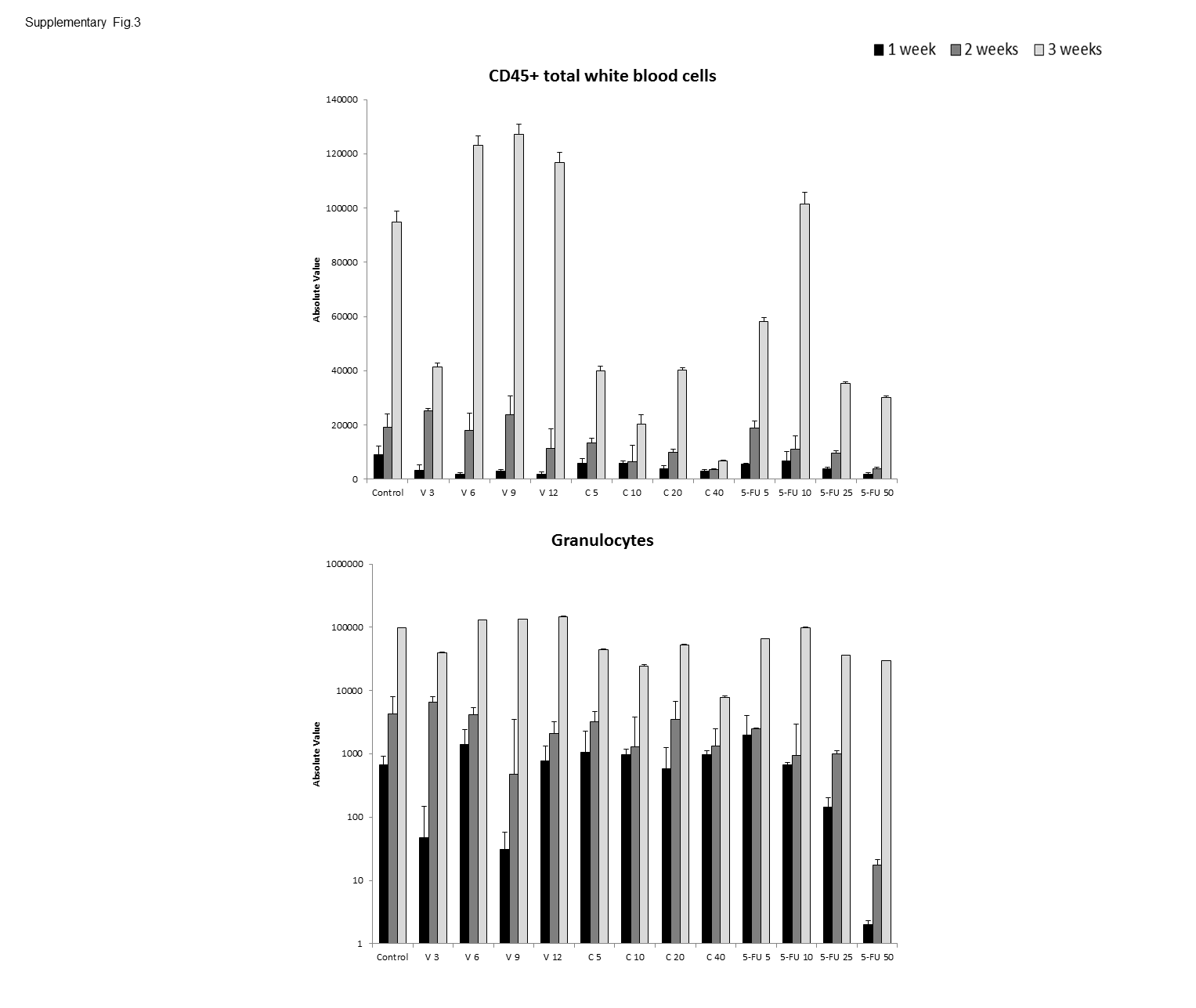

Supplement: Supplementary file 4 — Suppl. Fig. 3 [file 41416_2018_76_MOESM4_ESM.tif]

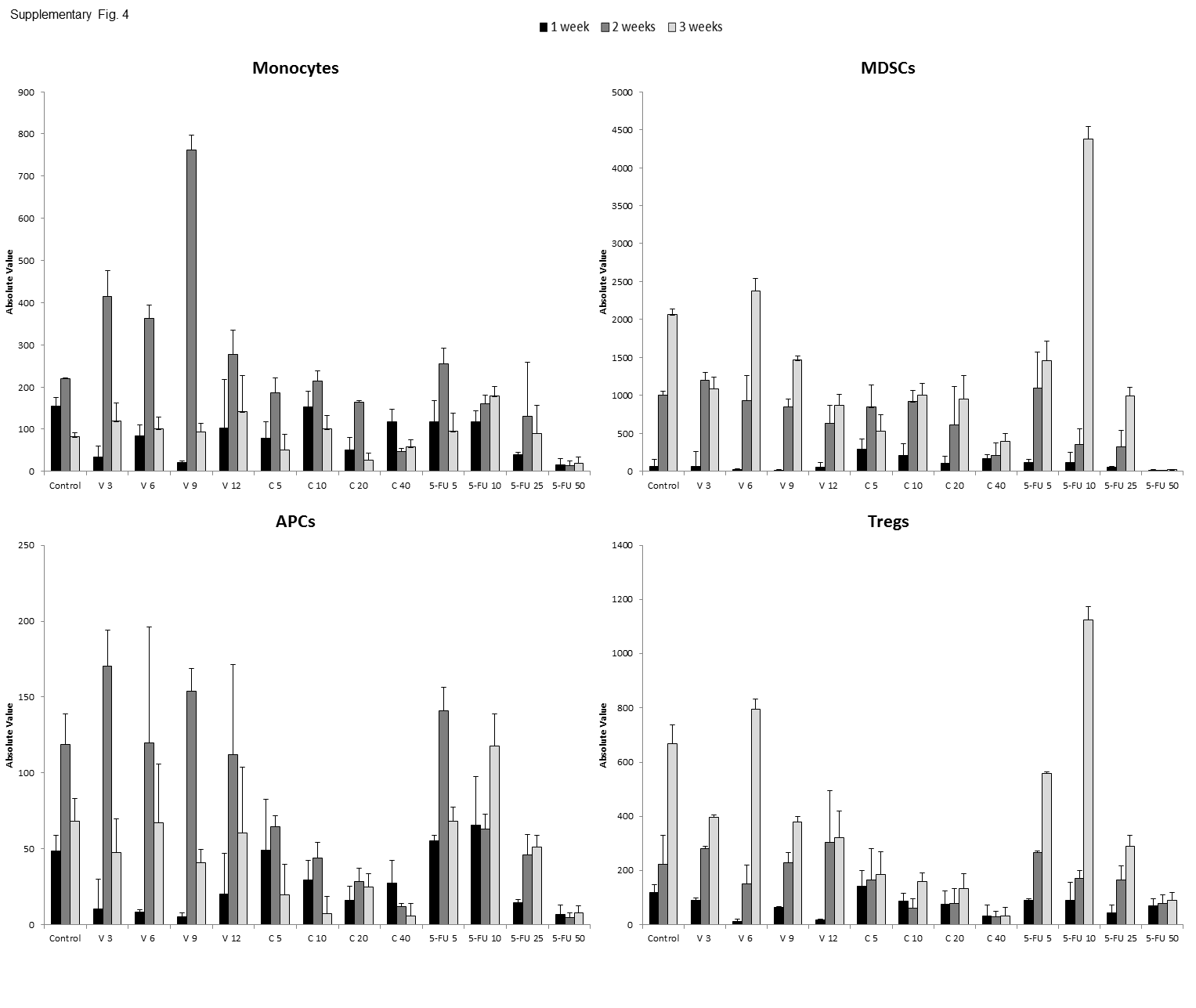

Supplement: Supplementary file 5 — Suppl. Fig. 4 [file 41416_2018_76_MOESM5_ESM.tif]

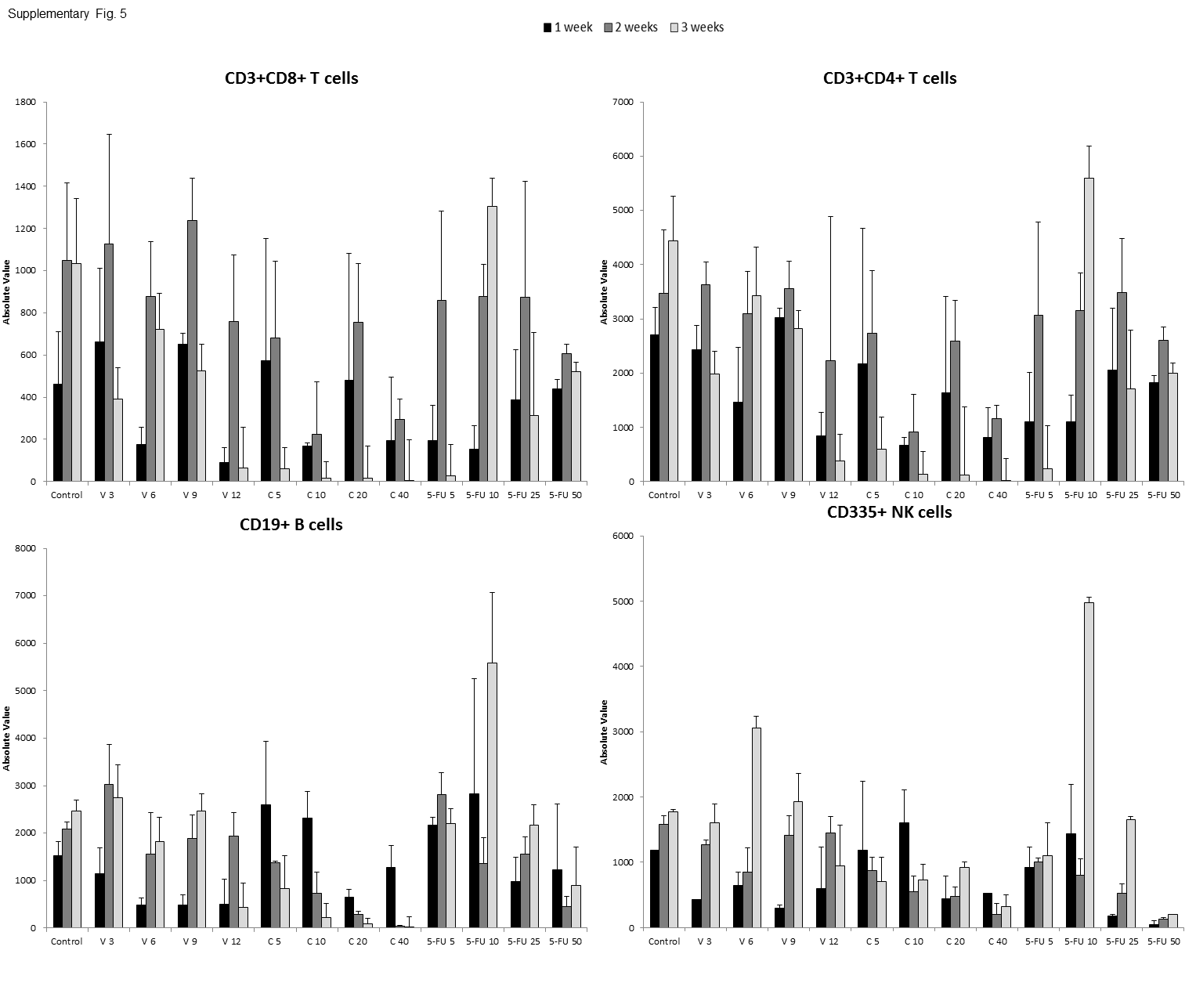

Supplement: Supplementary file 6 — Suppl. Fig. 5 [file 41416_2018_76_MOESM6_ESM.tif]

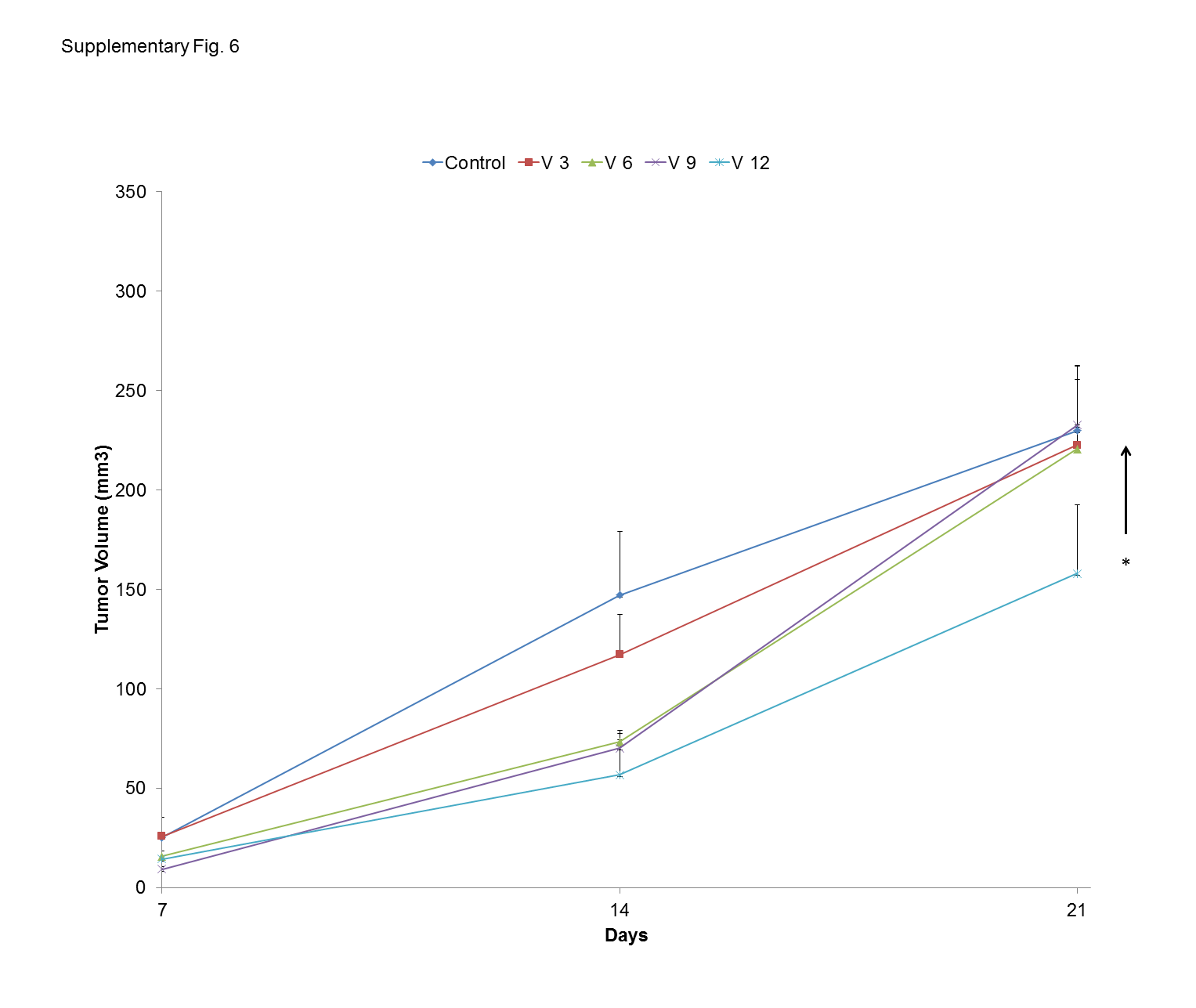

Supplement: Supplementary file 7 — Suppl. Fig. 6 [file 41416_2018_76_MOESM7_ESM.tif]

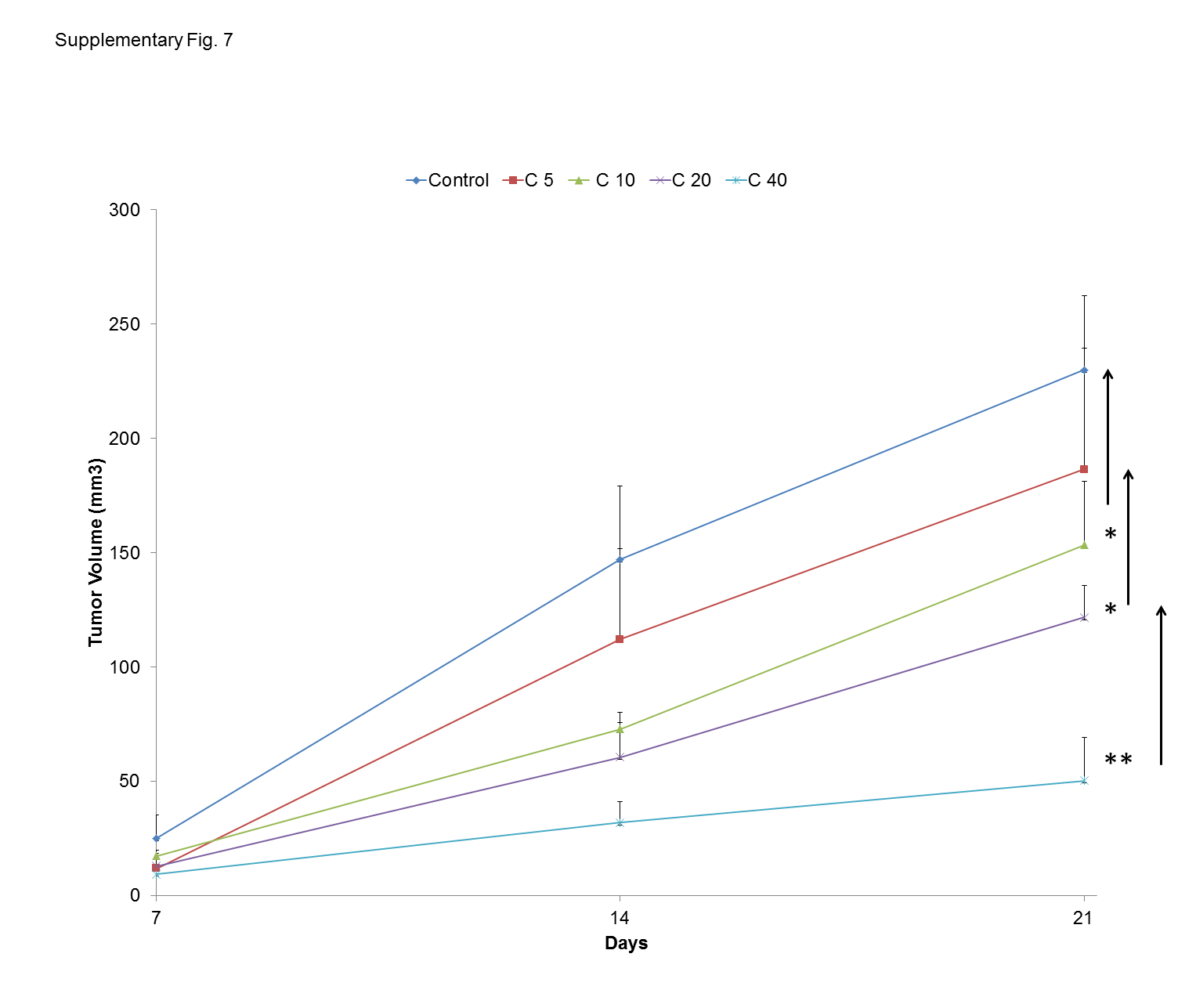

Supplement: Supplementary file 8 — Suppl. Fig. 7 [file 41416_2018_76_MOESM8_ESM.tif]

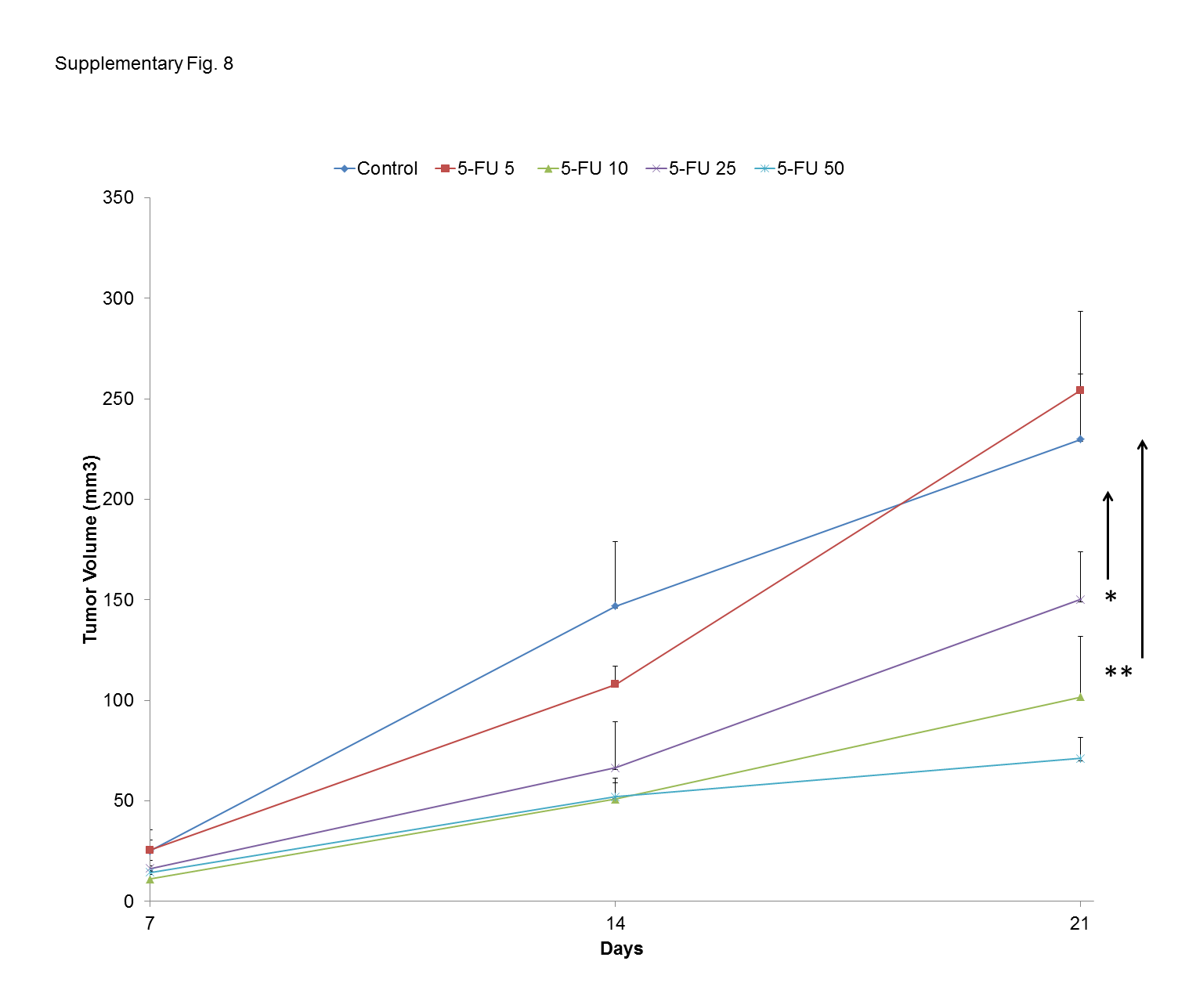

Supplement: Supplementary file 9 — Suppl. Fig. 8 [file 41416_2018_76_MOESM9_ESM.tif]

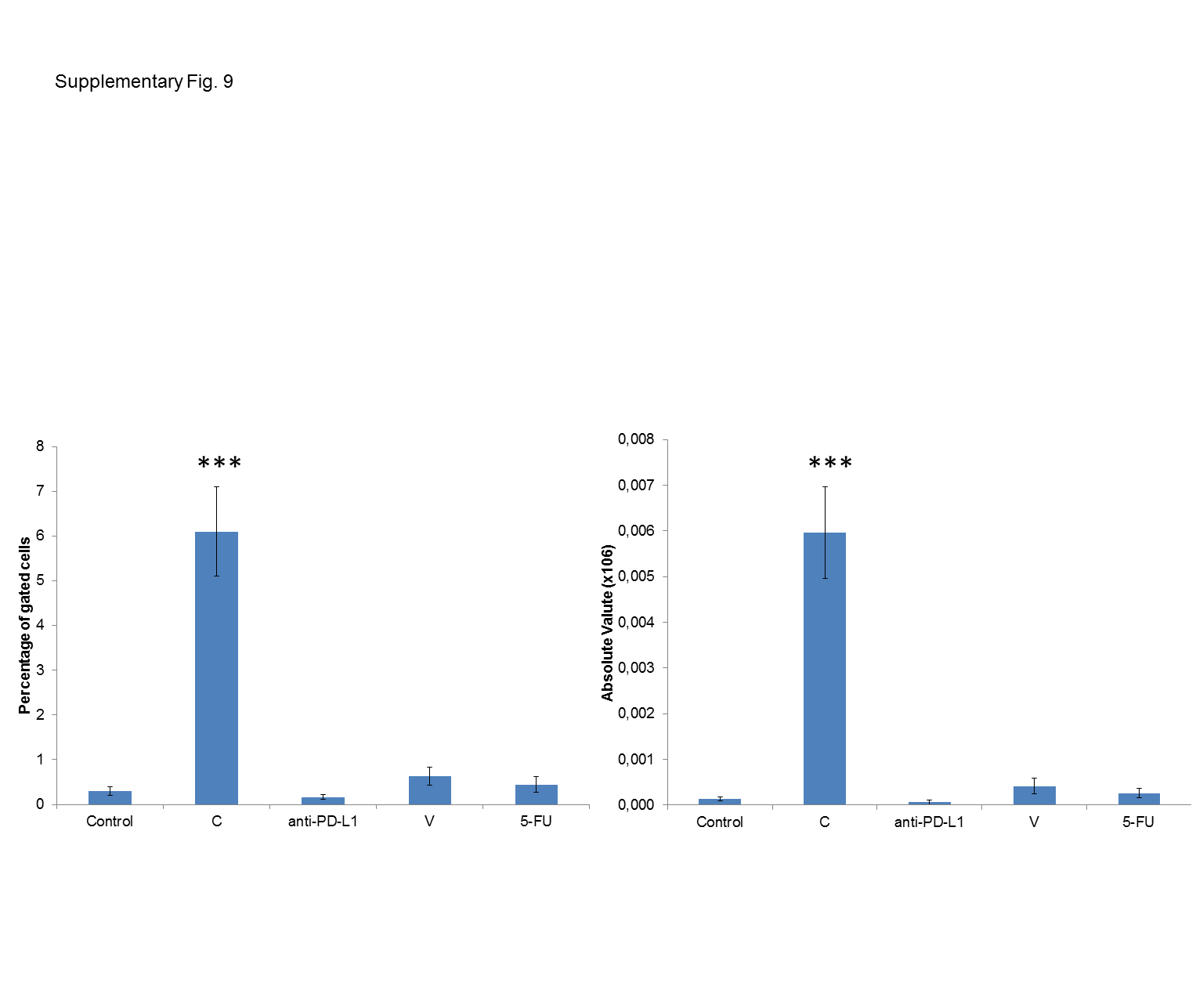

Supplement: Supplementary file 10 — Suppl. Fig. 9 [file 41416_2018_76_MOESM10_ESM.tif]

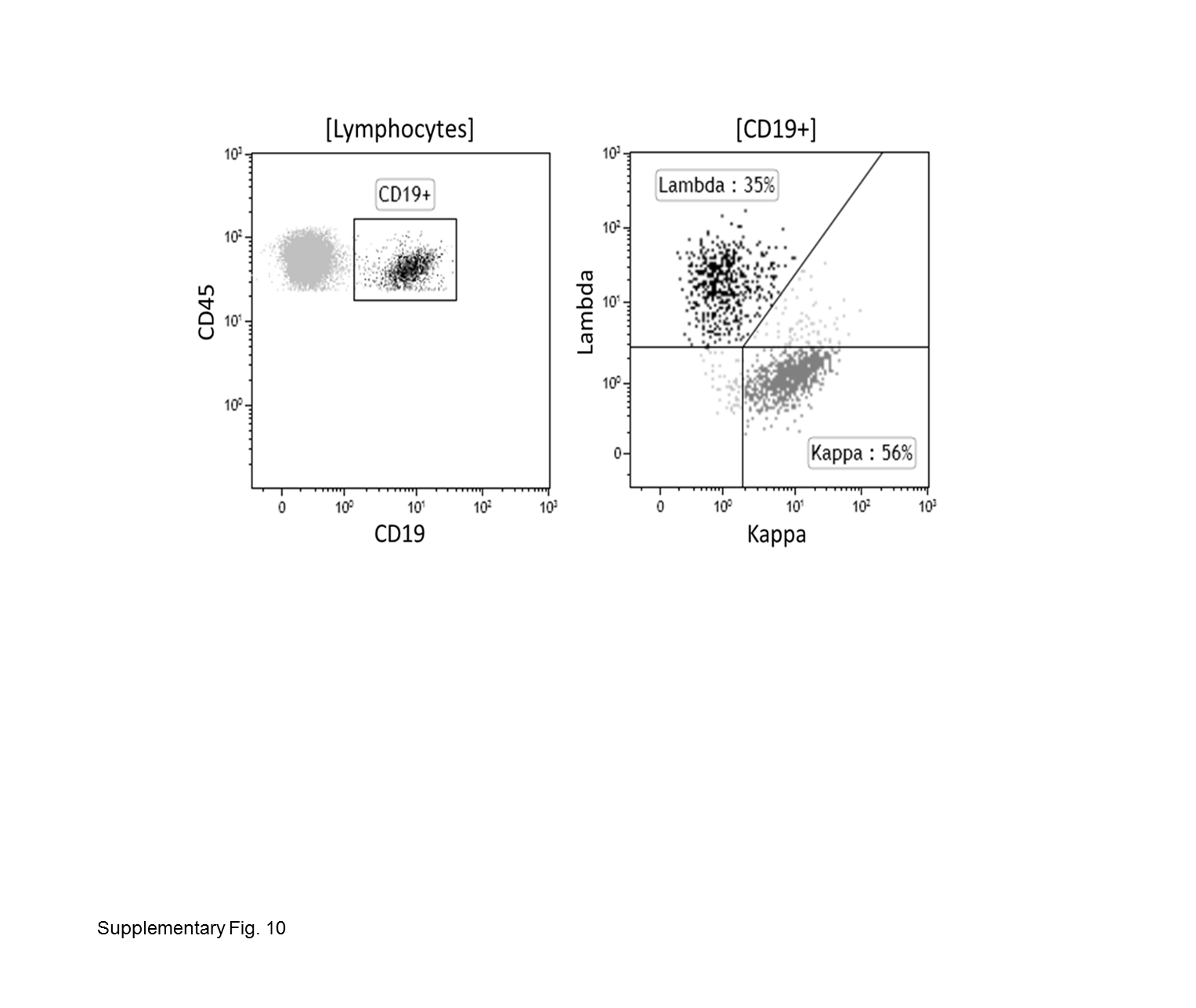

Supplement: Supplementary file 11 — Suppl. Fig. 10 [file 41416_2018_76_MOESM11_ESM.tif]

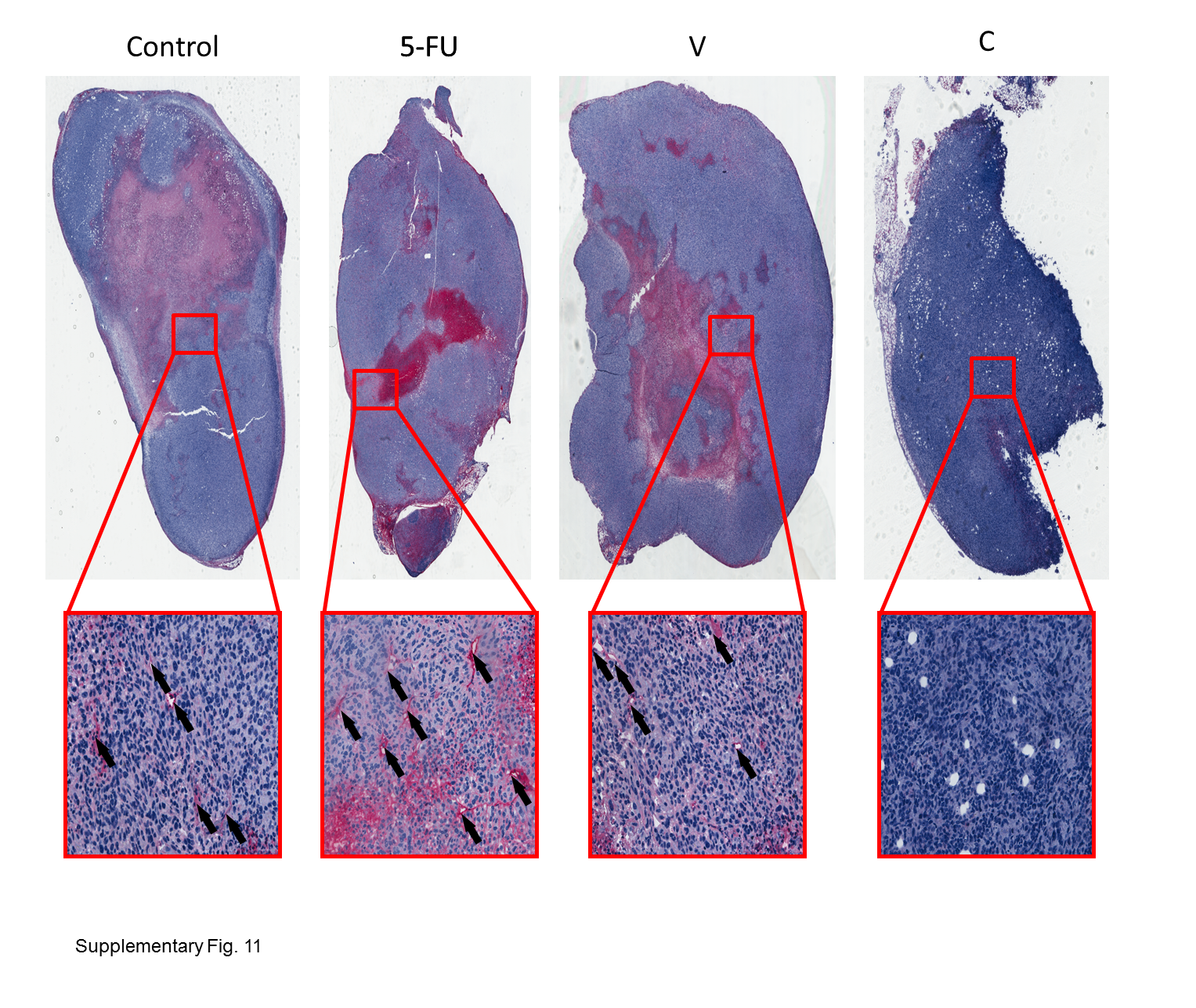

Supplement: Supplementary file 12 — Suppl. Fig. 11 [file 41416_2018_76_MOESM12_ESM.tif]

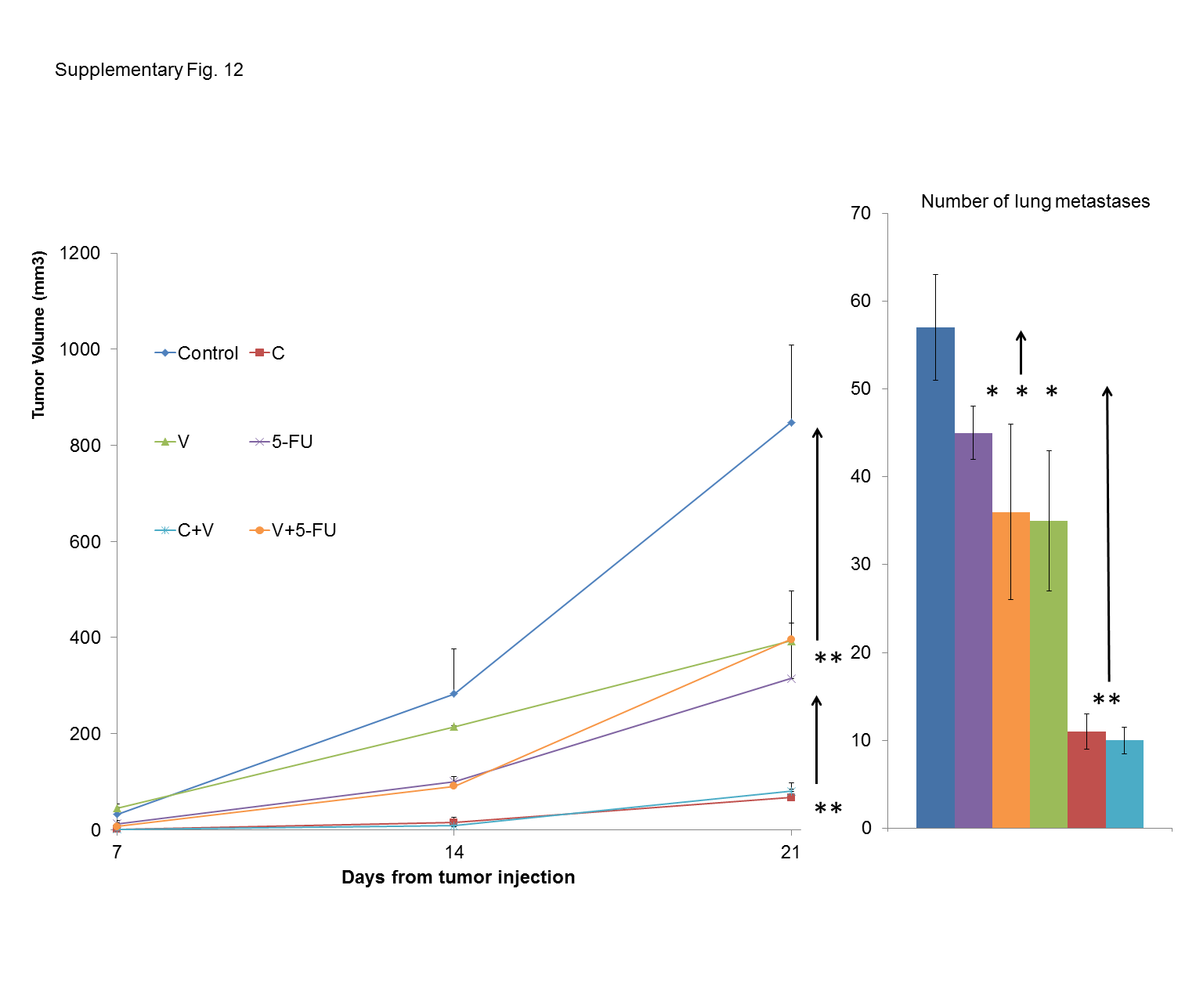

Supplement: Supplementary file 13 — Suppl. Fig. 12 [file 41416_2018_76_MOESM13_ESM.tif]

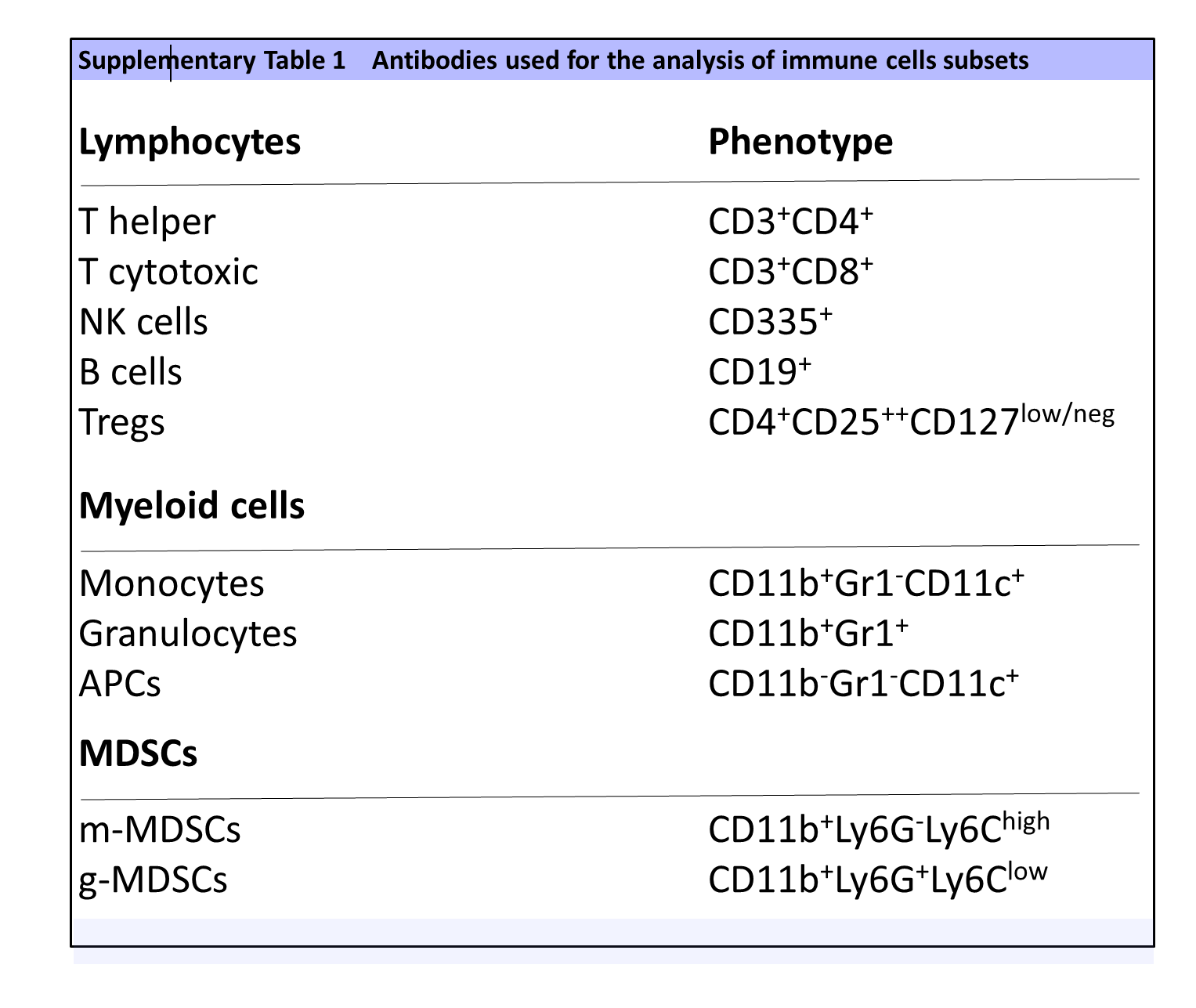

Supplement: Supplementary file 14 — Suppl. Table 1 [file 41416_2018_76_MOESM14_ESM.tif]

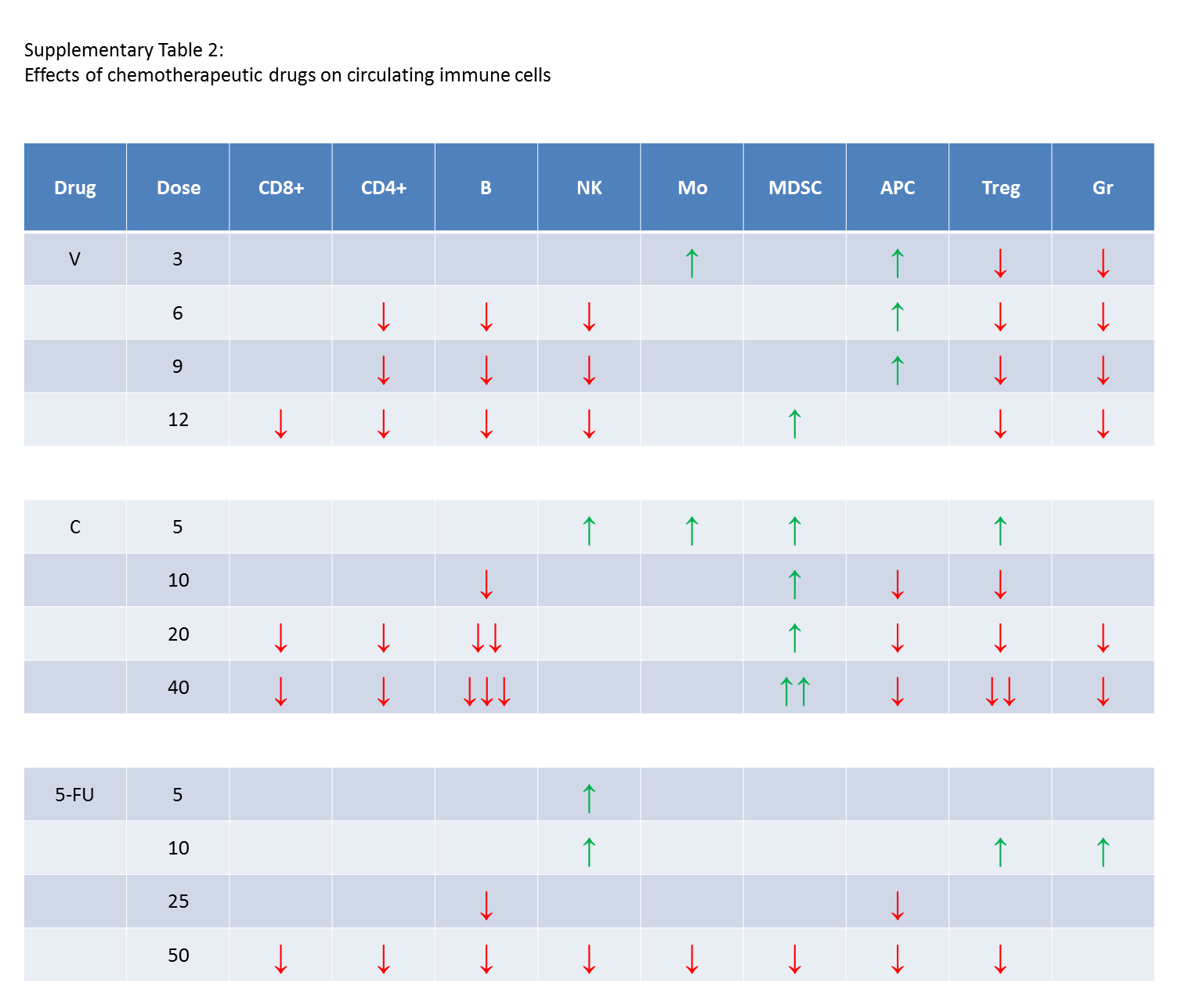

Supplement: Supplementary file 15 — Suppl. Table 2 [file 41416_2018_76_MOESM15_ESM.tif]
